# PATTERN OF CUTANEOUS MANIFESTATIONS IN DIABETES MELLITUS

**DOI:** 10.4103/0019-5154.60349

**Published:** 2010

**Authors:** Abhishek Goyal, Sujeet Raina, Satinder S Kaushal, Vikram Mahajan, Nand Lal Sharma

**Affiliations:** *From the Department of Medicine, Indira Gandhi Medical College, Shimla - 171 001, HP, India.*; 1 *From the Department of Dermatology, Indira Gandhi Medical College, Shimla - 171 001, HP, India.*

**Keywords:** *Diabetes mellitus*, *skin lesions*, *Western Himalayas*

## Abstract

**Background:**

Diabetes mellitus affects individuals of all ages and socioeconomic status. Skin is affected by the acute metabolic derangements as well as by chronic degenerative complications of diabetes.

**Aims:**

To evaluate the prevalence of skin manifestations in patients with diabetes mellitus. To analyze the prevalence and pattern of skin disorders among diabetic patients from this region of Western Himalayas.

**Materials and Methods:**

One hundred consecutive patients with the diagnosis of diabetes mellitus and having skin lesions, either attending the diabetic clinic or admitted in medical wards were included in this study.

**Results:**

The common skin disorders were: Xerosis (44%), diabetic dermopathy (36%), skin tags (32%), cutaneous infections (31%), and seborrheic keratosis (30%).

**Conclusion:**

Skin is involved in diabetes quite often and the manifestations are numerous. High prevalence of xerosis in our diabetic population is perhaps due to cold and dry climatic conditions in the region for most of the time in the year.

## Introduction

Diabetes mellitus affects individuals of all ages and in all socio-economic segments of the population. Global presence of type 2 diabetics in the year 2000 was 171 million which is likely to be 366 million in the year 2030.[1] The International Diabetes Federation (IDF) estimates the total number of diabetic subjects to be around 40.9 million in India and this is further set to raise to 69.9 million by the year 2025.[2] Estimates by WHO suggest that the number of diabetic subjects would increase to 80 million by the year 2030 in India.[1] Skin lesions are frequently observed in diabetic patients and about 30% of diabetics have cutaneous disorders.[3] The skin is affected by the acute metabolic derangements and the chronic degenerative complications of diabetes. Although the mechanism for many diabetes-associated skin conditions remains unknown, the pathogenesis of others is linked to abnormal carbohydrate metabolism, other altered metabolic pathways, atherosclerosis, microangiopathy, neuron degeneration, and impaired host mechanisms.[4] Only a few epidemiologic studies have been done on the prevalence of skin disorders in patients with diabetes mellitus.[3,5] There are no epidemiologic data related to skin disorders in diabetics reported from the Northern state of Himachal Pradesh, India. This study was designed to analyze the prevalence and pattern of skin disorders among diabetic patients from this region of Western Himalayas.

## Materials and Methods

The study was conducted in the Departments of Medicine and Dermatology of IGMC, Shimla. One hundred consecutive patients with the diagnosis of diabetes mellitus and having skin lesions, either attending the diabetic clinic or admitted in medical wards constituted the study population. Clinical details regarding age, sex, duration of diabetes mellitus, and treatment modalities were noted. All the patients underwent a detailed dermatological examination. Relevant microbiological and histopathological investigations to confirm the diagnosis were carried out.

## Results

The study comprised of 100 consecutive patients of diabetes mellitus with skin lesions. There were 54 males and 46 females (M:F = 1.7:1). The youngest patient was 28 years and oldest was 80 years with a mean age of 57.44 ± 10.37 years. The duration of diabetes was <10 years in 60 patients. Thirty four patients had 11-20 years of diabetes, and six had >20 years of diabetes. Ten patients were newly diagnosed as diabetics.

Various types of skin lesions and duration of diabetes mellitus observed are presented in Table 1. Various types of skin infections observed are shown in Table 2]. Majority of patients (80%) had combination of more than one type of skin lesions. Twenty patients had two types of skin lesions, 12 had three types, sixteen had four types, and another 16 had five types. Six types of skin lesions were observed in another 14 patients and only two patients presented with a maximum of seven. Twenty patients had only a single type of skin lesion.

**Table 1:** Type of skin lesions observed in diabetes mellitus

Types of skin lesions	Number of patients
Xerosis	44
Diabetic dermopathy	36
Skin tags	32
Infections	31
Seborrheic keratosis	30
Nail changes	20
Xanthalasma	10
Ichthyosis	10
Acanthosis nigricans	8
Vitiligo	8
Scleroderma-like skin	6
Diabetic rubeosis	4
Scabies	4
Lipodystrophy	4
Macular amyloidosis	4
Nodular prurigo	4
Diabetic bullae	2
Alopecia	2
Plantar psoriasis	2
Angiomatosis	2
Rosacea	2
Asteatotic eczema	2
Keloid	2
Nevi	2
Photodermatitis	2

**Table 2:** Type of skin infection in diabetes mellitus

Type of skin infection	Number of patients
Bacterial infection	
Impetigo contagiosa	4
Boils	6
Erythrasma	2
Folliculitis	3
Fungal infection	
Candidal	9
Dermatophytosis	7

## Discussion

Cutaneous signs of diabetes mellitus are extremely valuable to the clinician. They generally appear after the primary disease has developed but may signal or appear coincidentally with its onset, or even precede diabetes by many years.

Cutaneous manifestations of diabetes are classified into four categories: Skin lesions with strong-to-weak association with diabetes (necrobiosis lipiodica, diabetic dermopathy, diabetic bullae, yellow skin, eruptive xanthomas, perforating disorders, acanthosis nigricans, oral leucoplakia, lichen planus), infections (bacterial, fungal), cutaneous manifestations of diabetic complications (microangiopathy, macroangiopathy, neuropathy), and skin reactions to diabetic treatment (sulphonylureas or insulin).[3] Most documented studies have shown the incidence of cutaneous disorders associated with diabetes to be between 30 and 71%.[3,6] In our study, the most common six skin disorders were: Xerosis (44%), diabetic dermopathy (36%), skin tags (32%), cutaneous infections (31%), and pruritis and seborrheic keratosis −30% each, respectively. Xerosis accounted for the most common skin manifestation in our study and 44% patients had xerosis although various studies on cutaneous lesions in diabetic patients do not comment on the prevalence of xerosis. The reason for high prevalence of xerosis in our diabetic population is perhaps due to cold and dry climatic conditions in the region for most of the time in the year. Diabetic dermopathy, in the form of small, atrophic, brown-scar-like macules on both chins were seen in 36% of the patients. Diabetic dermopathy may develop from the factors that lead to the development of vascular complications of diabetes and it may serve as a clinical sign of an increased likelihood of vascular complications in diabetic patients.

Skin tags were seen in 32% of patients. Skin tags may serve as a marker for diabetes mellitus as was concluded by Thappa *et al*.[7] Cutaneous infections were seen in 31% of patients. Fungal infections were seen in 16% of the patients (9% had candidal and 7% had dermatophytosis). Bacterial infections were seen in 15% of the patients. It is widely believed that diabetic patients have an increased risk for infectious diseases, although there is little documented evidence to support it. This risk seems to be higher in poorly controlled patients, but it is often difficult to understand whether poor metabolic control is the cause or the consequence of the concurrent infections.[4] None of the patients had viral infections, wet gangrene, scleroderma diabeticorum, trophic ulcer, granuloma annulare, necrobiosis lipiodica, lichen planus, reactive perforating collagenosis, or drug reactions to oral hypoglycemics in this study, although these are usually associated with diabetes mellitus.

From the foregoing account, we conclude that the skin is involved in diabetes quite often. The manifestations are numerous and varied and many a times they can serve as diagnostic marker for underlying diabetes. Whenever patients present with multiple skin manifestations, their diabetic status should be checked. The recognition of these skin findings is the key to treatment and prevention.
